# Conjunctival Limbal Autograft Combined with Amnion-Assisted Conjunctival Epithelial Redirection for Unilateral Total Limbal Stem Cell Deficiency after Severe Chemical Burn

**DOI:** 10.3390/jcm12196235

**Published:** 2023-09-27

**Authors:** Tian-Yu Yao, Jia-Song Wang, Wen Geng, Hua-Tao Xie, Ming-Chang Zhang

**Affiliations:** Department of Ophthalmology, Union Hospital, Tongji Medical College, Huazhong University of Science and Technology, Wuhan 430022, China; yaotianyu@hust.edu.cn (T.-Y.Y.); jiasongwang@hust.edu.cn (J.-S.W.); gengwen0620@163.com (W.G.)

**Keywords:** amniotic membrane, chemical burn, conjunctival epithelial redirection, limbal autograft, limbal stem cell deficiency

## Abstract

(1) Background: To evaluate the efficacy of conjunctival limbal autograft (CLAU) combined with the amnion-assisted conjunctival epithelial redirection (ACER) procedure for patients with unilateral total limbal stem cell deficiency (LSCD) caused by severe chemical burn. (2) Methods: A retrospective interventional case series of unilateral total LSCD after chemical burn who underwent CLAU combined with ACER surgery between September 2021 and July 2023 was collected. Outcome measures included epithelialization of the cornea with donor limbus-derived epithelium, best corrected visual acuity (BCVA), and complications. (3) Results: Nine males and one female were included in this study. The mean age was 40.9 ± 9.63 (range, 26 to 55) years. The average duration between injury and CLAU combined with the ACER procedure was 7.67 ± 3.97 (range, 4 to 18) months. All patients achieved corneal epithelialization and improved BCVA. Postoperative complications occurred in four cases, including delayed corneal epithelial healing in one case, delayed amniotic membrane dissolution and detachment in two cases, and recurrence of symblepharon in one case. No complications were noted in the healthy donor eyes. (4) Conclusions: CLAU combined with ACER is a safe and effective treatment for unilateral total LSCD caused by severe chemical burn. This combined surgery restores visual function for patients with corneal blindness caused by chemical burn, reducing the burden on the families and society.

## 1. Introduction

Limbal stem cells are adult somatic stem cells located at the limbus, a niche area between the cornea and conjunctiva representing the source of transparent and intact corneal epithelium [1]. Ocular chemical burn is one of severe ocular surface injuries, especially in developing countries, which brings a heavy economic burden on families and society [2]. After first-stage irrigation and anti-inflammatory treatment, severe ocular surface chemical damage often develops into total limbal stem cell deficiency (LSCD), which leads to conjunctivalization, neovascularization, and corneal scarring, resulting in blindness [3].

Conjunctival limbal autograft (CLAU) is the most commonly used surgical procedure for unilateral LSCD [4,5], but it is restricted by the limited size of autologous limbal tissue. Highly proliferative conjunctival cells are prone to re-invade the cornea from areas not covered by grafts. The clinical efficacy of autologous simple limbal epithelial transplantation (SLET) has been validated for monocular LSCD; however, SLET alone is not effective in cases with severe symblepharon [6]. Cultivated autologous limbal epithelial transplantation necessitated complex manufacturing procedures compatible with good manufacturing practices and is only applied in hospitals with excellent laboratories [7,8,9]. Dua et al. proposed an CLAU with the amnion-assisted conjunctival epithelial redirection (ACER) procedure, which utilized the amniotic membrane (AM) to redirect the conjunctival epithelial hyperplasia and thus was able to achieve the goal of grafted limbal epithelial cell proliferation to complete corneal epithelialization [10,11].

However, the efficacy of this procedure for total LSCD caused by chemical burn, especially for those cases with complete conjunctivalization and severe symblepharon, is still unknown. Herein, we share our experiences from the combination application of CLAU and ACER in the treatment of unilateral total LSCD after severe chemical burn with or without symblepharon.

## 2. Materials and Methods

This retrospective study was approved by the Ethics Committee of Union Hospital, Tongji Medical College, Huazhong University of Science and Technology, according to the Declaration of Helsinki (UHCT230505). Medical records from consecutive patients from September 2021 to July 2023 with unilateral total LSCD after chemical burn who underwent CLAU combined with ACER surgery were collected.

### 2.1. Participants

All patients who underwent surgery were diagnosed with severe total LSCD, and the inclusive criteria were as follows: (1) a clear history of ocular surface chemical burn; (2) diagnosis of Grade IV burn [12]: conjunctiva formation on the whole corneal limbus was seen; (3) acceptance of CLAU and ACER treatment.

Demographic information, complete medical history in the hospital, corneal epithelization time, AM detaching time, best corrected visual acuity (BCVA) before surgery and among follow-up, and the last follow-up time were recorded. The BCVA was determined by the Standard Logarithm Visual Acuity Chart. At the same time, each patient’s preoperative and postoperative ophthalmic examination data were also preserved in detail, including slit-lamp observation records, anterior segment photography, and corneal optical coherence tomography.

### 2.2. Surgical Technique

All surgeries were performed by the same ophthalmologist at Union Hospital, Tongji Medical College, Huazhong University of Science and Technology.

**Figure 1:**
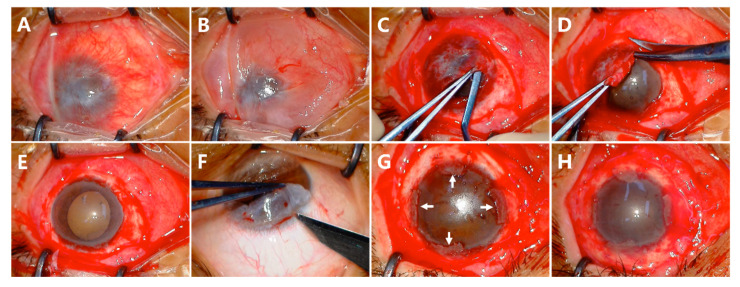
Surgical steps of CLAU combined with ACER. The cornea and scarred conjunctival tissue were exposed (**A**). Conjunctival tissues were separated from the cornea and sclera by the injection of lidocaine (**B**). The fibrovascular pannus on the cornea was dissected by a blunt scraper (**C**) and peeled off (**D**). The limbus was exposed, and the fornix was formed (**E**). The conjunctival limbal graft of the 4 clock hour was taken from the contralateral eye (**F**). The graft was then sutured onto the limbus at 3, 6, 9, and 12 o’clock (**G**). A piece of amniotic membrane (AM) was taken to cover the corneal surface with the epithelial face up, and the conjunctival edge, AM, and the superficial sclera were fixed with 8-0 absorbable suture interuptly (**H**).

After the cornea and peripheral conjunctiva were adequately exposed (Figure 1A), subconjunctival 2% lidocaine was injected to separate the conjunctiva from the cornea and sclera (Figure 1B). After removing the scar tissue underneath, the conjunctiva was preserved as much as possible (Figure 1C). The fibrovascular pannus on the cornea was dissected by a blunt scraper (Figure 1C) and peeled off (Figure 1D). The released conjunctival tissue was used to form a normal depth of fornix (Figure 1E). The conjunctival limbal graft of the 4 clock hour was taken from the contralateral healthy eye, including at least 1 mm of superior limbus and 3 mm of adjacent conjunctiva (Figure 1F). The graft was marked superiorly and inferiorly on the conjunctival side. The graft was then sutured onto the limbus and fixed to the superficial sclera with 8-0 absorbable sutures at 3, 6, 9, and 12 o’clock, individually (Figure 1G). A piece of AM with the epithelium side up was applied to cover the cornea, and the edge of the membrane was tucked under the recessed conjunctival edge. The conjunctival edge, AM, and the superficial sclera were fixed with 8-0 absorbable suture interuptly (Figure 1H). The whole surgical process was recorded in the Supplementary Video S1.

### 2.3. Postoperative Treatment and Follow-Up

After surgery, all eyes were treated with Tobradex eye drops (tobramycin 0.3% and dexamethasone 0.1%, Alcon, Fort Worth, TX, USA) 4 times per day, Tobradex eye ointment (tobramycin 0.3% and dexamethasone 0.1%, Alcon, Fort Worth, TX, USA) once nightly for 1 month, and then 0.1% fluorometholone eye drops (Allergan, County Mayo, Ireland) 4 times per day followed by a weekly taper for 4 weeks. The duration and frequency of instillation were also adjusted depending on the degree of inflammation. Diquas eye drops (diquafosol 3%, Santen, Japan) were administrated 4 times per day to moisten the ocular surface for 4–8 weeks. After the conjunctival edema regressed, bandage contact lenses (Bausch Lomb, Laval, QC, USA) were used for 4 weeks to reduce the foreign body sensation and stabilize the AM. All patients were followed up daily for 1 week, weekly for 1 month, monthly for 3 months, and then at different intervals after surgery.

### 2.4. Outcome Measures

The primary outcome measure was complete epithelialization of the cornea with a donor limbus-derived epithelium and the prevention of the conjunctival epithelium from encroaching on the corneal surface. The second outcome measurement was BCVA improvement. Complication surveys included delayed corneal epithelial healing, recurrence of symblepharon, delayed AM dissolution and detachment, and limbal stem cell deficiency in the donor eyes.

## 3. Results

### 3.1. Pretreatment Characteristics

Demographics of this study population are presented in Table 1. Nine males (90%) and one female (10%) were included in this study. The mean age was 40.9 ± 9.63 (range, 26 to 55) years. A total of six right eyes and four left eyes were included. All patients had a definite history of chemical injuries, including eight patients with alkali burns, one patient with exposure to acidic chemicals, and one patient with hair dye. Seven patients (70%) were diagnosed with symblepharon. The average time of duration between injury and CLAU combined with ACER procedure was 7.67 ± 3.97 months (range, 4 to 18 months).

### 3.2. Conjunctival Epithelial Redirection

**Figure 2:**
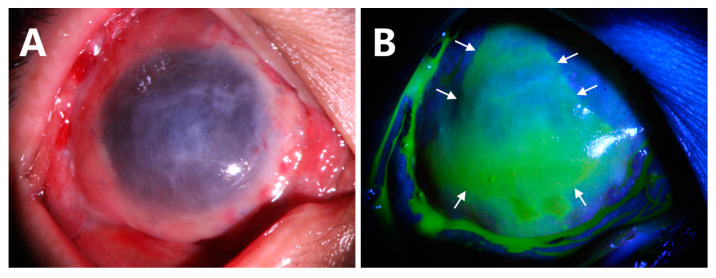
AM was closely attached to the cornea (**A**). Fluorescein staining showing the conjunctival epithelial cells were redirected to AM ((**B**), white arrows).

The conjunctival epithelium from the recessed conjunctiva could be clearly seen to migrate on the AM at the third day after operation, indicating that the proliferating conjunctival epithelial cells were successfully redirected (Figure 2B, white arrows). The AM was found to be completely epithelialized by conjunctival epithelial cells at week 4 of the follow-up visit.

### 3.3. Corneal Epithelialization in Patients without Symblepharon

**Figure 3:**
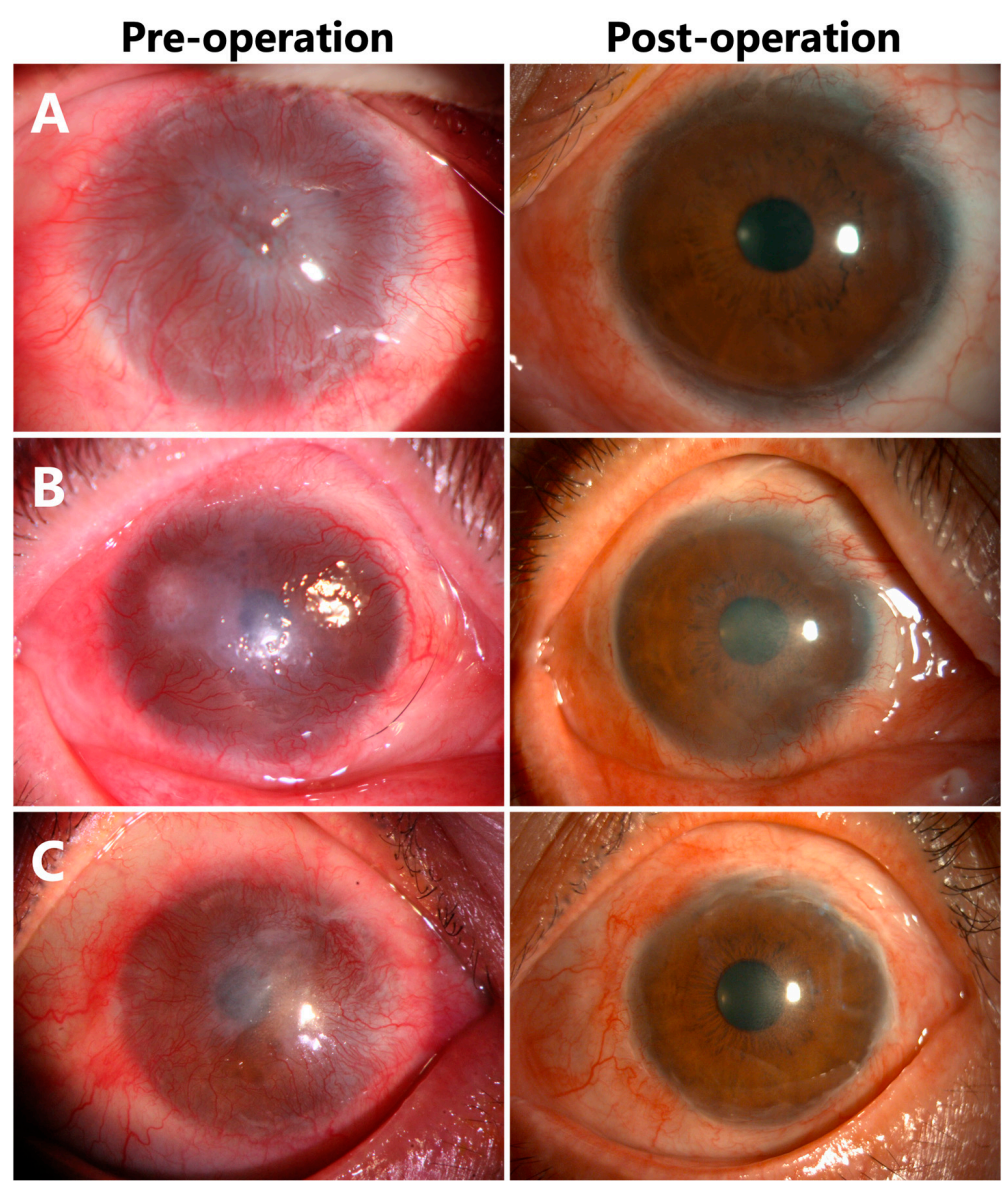
Corneal epithelialization in Case 1 (**A**), Case 2 (**B**), and Case 5 (**C**) without symblepharon.

Three eyes (30%) (Case 1, 2, 5) were diagnosed with total LSCD without symblepharon (Figure 3A–C). Epithelialization of the corneas were completed with donor limbus-derived epithelium without any conjunctival epithelium in all three cases. BCVA was elevated from HM to 1.0 in Cases 1 and 5 (Figure 3A,C), and from CF to 0.3 in Case 2 (Figure 3B). No complications were noted during the follow-up period (21 months in Case 1, 19 months in Case 2, 7 months in Case 5).

### 3.4. Corneal Epithelialization in Patients with Symblepharon

**Figure 4:**
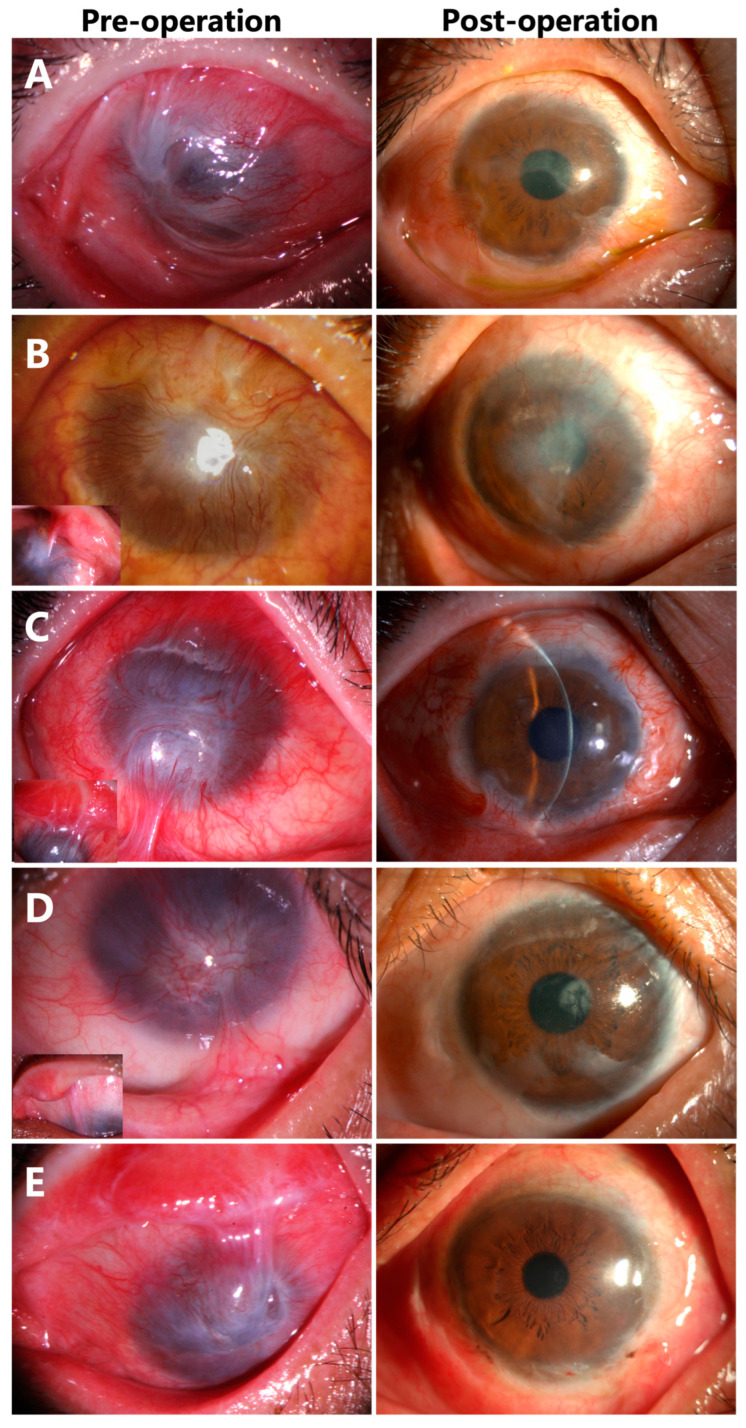
Corneal epithelialization in Case 3 (**A**), Case 4 (**B**), Case 6 (**C**), Case 7 (**D**), and Case 8 (**E**) with symblepharon.

Seven eyes (70%) were diagnosed with total LSCD and symblepharon (Figure 4A–E, inserts). Epithelialization of the corneas were completed with donor limbus-derived epithelium without conjunctival epithelium in all seven cases at the last follow-up visit. Although corneal opacity was noticed in the pupil area in Case 7, the BCVA recovered from HM/10 cm to 0.8 (Figure 4D).

### 3.5. Visual Acuity

The mean follow-up period was 12.3 ± 5.5 (range 6–21) months. All patients had improved BCVA at the last follow-up visit (Table 2).

### 3.6. Complications

**Figure 5:**
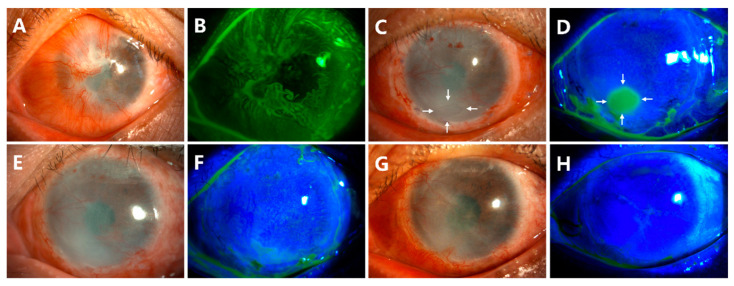
Delayed corneal epithelialization in Case 10. The neovascular membrane grew into the cornea and was severe on the nasal side preoperatively (**A**,**B**). A corneal epithelial defect was noted at week 4 of the follow-up visit ((**C**,**D)**, white arrows). The corneal epithelialization completed after 2 weeks’ treatment (**E**,**F**). At the follow-up of month 18, the ocular surface remained smooth and stable (**G**,**H**).

**Figure 6:**
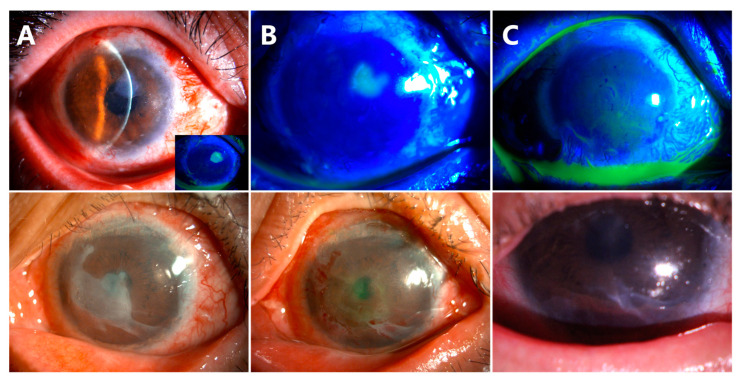
Delayed AM dissolution and detachment. Small pieces of AM were found on the corneal surface (**A**). Partial corneal epithelial defects were observed after scraping off AM (**B**). The central corneas were smooth and transparent at week 10 follow-up (**C**).

**Figure 7:**
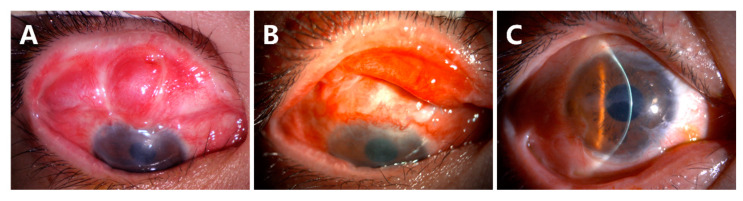
Recurrence of symblepharon in Case 3. The disappearance of the superior conjunctival fornix was noted (**A**). The superior fornix was well formed at 2 months after AM transplantation (**B**). The ocular surface was stable at the follow-up visit of 13 months (**C**).

(1) Delayed corneal epithelial healing occurred in Case 10 (Figure 5). The preoperative anterior segment photograph showed that the pannus had grown into the cornea from 360 degrees (Figure 5A,B). At the follow-up of week 4 after surgery, there was a corneal epithelial defect of approximately 2 × 3 mm^2^ (Figure 5C,D, white arrows). The patient was treated with soft contact bandage lenses. The corneal epithelization was completed 2 weeks later (Figure 5E,F). The ocular surface remained smooth and stable at the last follow-up of month 18 (Figure 5G,H).

(2) Delayed AM dissolution and detachment occurred in Cases 6 (Figure 6, the first row) and 7 (Figure 6, the second row). At the follow-up visit of week 8, a small piece of AM was still attached on the corneal surface (Figure 6A). The residual AM was scraped off in both patients (Figure 6B). The central corneas were smooth and transparent at the follow-up visit of week 10 (Figure 6C).

(3) Recurrence of symblepharon occurred in Case 3 (Figure 7). This patient had symblepharon before surgery. Eight weeks after CLAU combined with the ACER procedure, the corneal epithelialization was completed, but symblepharon recurred as presented by the superior conjunctival fornix disappearance (Figure 7A), and trichiasis was also complicated. Entropion, trichiasis, and symblepharon correction were performed. AM transplantation was used to form the conjunctival fornix. The postoperative follow-up of 2 months showed that the superior fornix was well formed (Figure 7B). The ocular surface was stable and smooth without any complications at the last follow-up of 13 months (Figure 7C).

(4) For all 10 subjects undergoing biopsy, the donor eye healed without complications, and the visual acuity returned to baseline within 4 weeks.

## 4. Discussion

In this study, we present a modified CLAU combined with ACER procedures in managing unilateral total LSCD after severe chemical burn. Our results demonstrated that all the patients achieved a complete epithelialization of the cornea with a donor limbus-derived epithelium without the conjunctival epithelium encroaching onto the corneal surface. The BCVA improved significantly in all patients, although three patients needed further deep anterior lamellar keratoplasty due to severe corneal stromal opacity.

The limbus contains a population of self-renewing stem cells called limbal stem cells that are responsible for the maintenance of the integrity of the corneal surface and continuous renewal of the corneal epithelium [13]. In cases with complete loss of corneal and limbal epithelium with some surviving conjunctival epithelium after severe chemical injury, the conjunctival epithelium migrates centripetally to reach the limbus and covers the cornea [3,14]. Epithelialization of the cornea reduces the risk of melts and perforation because of persistent inflammation. Ex vivo-expanded sheets of the conjunctival epithelium [15] and oral mucosal epithelium [16] on AM have been used to reconstruct the corneal surface for epithelial defect in LSCD with acute inflammatory activity. Considering the precious value of autologous limbal tissue, ocular surface reconstruction using CLAU and ACER surgery should wait for the epithelialization of the ocular surface and the alleviation of inflammation to ensure the success of the surgery [17]. The conjunctival limbal graft of 4 clock hour was taken from the superior limbus of the contralateral healthy donor eye. The graft was then sutured onto the limbus at 3, 6, 9, and 12 o’clock, independently, in order to avoid the formation of pseudo-pterygium nasally and temporally. In the present study, the donor eye healed without complications, and the visual acuity returned to baseline within 4 weeks.

Cultivated autologous limbal epithelial transplantation is a significant advancement for ocular surface reconstruction [7,8,9]. In this approach, a tiny limbal biopsy from the healthy eye was used to create a multilayered sheet of corneal epithelium ready for transplantation. However, cell expansion necessitated a clinical-grade laboratory with regulatory approvals, which is extremely expensive to build and maintain. In 2012, Sangwan et al. described a SLET that allows limbal epithelial cells to expand on the recipient corneas [18]. SLET effectively restored a clear corneal surface with minimal neovascularization in patients with LSCD. Unilateral LSCD is the primary indication for autologous SLET. However, SLET alone is not effective in cases with severe symblepharon, which require additional conjunctival autografting, either during or after SLET [6]. SLET combined with ACER might be effective for these complicated cases. Another scenario where CLAU may hold advantage over SLET is in cases of complex reconstruction requiring conjunctival, limbal, and corneal grafting. In these cases, the corneal graft remains at high risk of immunological rejection and may need to be replaced in the future. Therefore, placing the limbal graft in its anatomical location beyond the cornea as in CLAU may be advantageous over SLET because the limbal transplants placed on the corneal graft will be lost when the corneal graft is replaced [6]. In the present study, 7 out of 10 patients had severe symblepharon. Six patients (85.7%) recovered with smooth ocular surface and resolution of symblepharon after CLAU and ACER. Only one patient (Case 3) had recurrence of symblepharon, which might have been due to postoperative cicatricial entropion and trichiasis [19].

**Figure 8:**
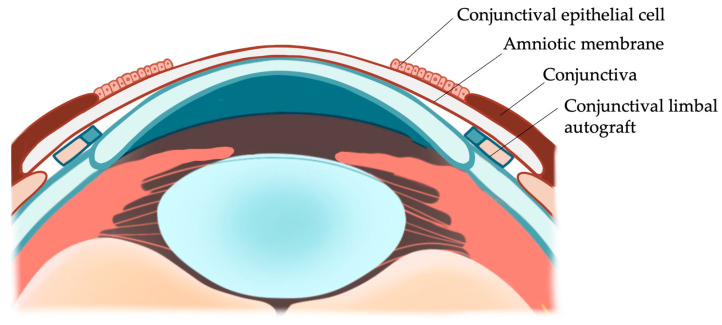
Graphical representation of CLAU combined with ACER. The conjunctival limbal grafts are sutured onto the limbus. A piece of AM with epithelium facing up covers the corneal surface and is tucked under the conjunctival edge. The conjunctival epithelial cells are redirected to the surface of the AM.

The AM has been used either as a graft [20] or combined as a graft and patch [21] in association with limbal transplantation. It is critical to ensure that the AM is tucked under the conjunctival edge in all quadrants in the modified ACER surgery. ACER keeps the conjunctival epithelium away from the corneal surface (Figure 8). ACER may also be applied in LSCD from various etiologies, such as bilateral chemical burn or Stevens–Johnson syndrome. This study was limited by the small sample size and retrospective observational design. Data were collected from patient records, so the quality of the dataset depended on the details of those records and was not standardized. Finally, this study evaluated patients from a single tertiary care center, which might limit outreach. Future comparative studies, ideally randomized controlled trials, comparing the effectiveness and safety of different techniques will help further inform the clinical practice.

## 5. Conclusions

CLAU combined with ACER is a safe and effective treatment for unilateral total LSCD caused by severe chemical burn. This combined surgery restores visual function for patients with corneal blindness caused by chemical burn, reducing the burden on the families and society.

## Figures and Tables

**Table 1:** Demographic and clinical features of patients who underwent the CLAU combined with ACER surgery: pre-operation.

Demographic and Clinical Features	Data
Age (Year)	
Mean	40.9 ± 9.63
Range	26–55
Gender	
Male	9 (90%)
Female	1 (10%)
Eye	
Right	6 (60%)
Left	4 (40%)
Chemical substances	
Alkali	8 (80%)
Nitric acid	1 (10%)
Hair dye	1 (10%)
Symblepharon	7 (70%)
Duration between injury and reconstruction (months)	
Mean	7.67 ± 3.97
Range	4–18

**Table 2:** Clinical data of patients who underwent the CLAU combined with ACER surgery.

Case Number	Symblepharon	Best Corrected Visual Acuity		Corneal Opacity	Complications	Other Surgeries	Follow-Up (Months)
		Pre- Operation	Last Visit				
1	No	HM/30 cm	1.0	No	No	No	21
2	No	CF/10 cm	0.3	Mild	No	No	19
3	Yes	HM/20 cm	0.15	Mild	Recurrence of symblepharon	Entropion correction and reconstruction of fornix	13
4	Yes	HM/10 cm	0.1	Severe	No	No	12
5	No	CF/20 cm	1.0	No	No	No	7
6	Yes	HM/20 cm	0.6	No	Delayed AM dissolution	AM scraping off	11
7	Yes	HM/10 cm	0.8	No	Delayed AM dissolution	AM scraping off	10
8	Yes	HM/10 cm	0.6	No	No	No	6
9	Yes	HM/30 cm	0.1	Severe	No	No	6
10	Yes	HM/10 cm	0.1	Severe	Delay of corneal epithelization	No	18

## Data Availability

The original contributions presented in the study are included in the article/Supplementary Material; further inquiries can be directed to the corresponding author.

## Supplementary Materials

The following supporting information can be downloaded at: https://www.mdpi.com/article/10.3390/jcm12196235/s1, Video S1: CLAU and ACER for LSCD.
